# Hybridization of molecules via a common photonic mode

**DOI:** 10.1073/pnas.2505161122

**Published:** 2025-07-30

**Authors:** Jahangir Nobakht, André Pscherer, Jan Renger, Stephan Götzinger, Vahid Sandoghdar

**Affiliations:** ^a^Nano-Optics Division, Max Planck Institute for the Science of Light, Erlangen D-91058, Germany; ^b^Department of Physics, Friedrich-Alexander University, Erlangen D-91058, Germany; ^c^Graduate School in Advanced Optical Technologies, Friedrich-Alexander University, Erlangen D-91052, Germany

**Keywords:** hybridization, polaritonic states, strong coupling, cavity quantum electrodynamics

## Abstract

Atoms and molecules usually hybridize and form bonds when they come in very close proximity of each other. In this work, we show that molecules can hybridize even through far-field electromagnetic interactions mediated by the shared mode of an optical microcavity. We discuss a collective enhancement of the vacuum Rabi splitting and study super- and subradiant states that arise from the cavity-mediated coupling both in the resonant and dispersive regimes. Moreover, we demonstrate a two-photon transition that emerges between the ground and excited states of the new optical compound. Our experimental data are in excellent agreement with the predictions of the Tavis–Cummings Hamiltonian and open the door to the realization of hybrid light–matter materials.

## Significance

Quantum mechanical interactions among atoms and molecules are commonly based on electromagnetic interactions between them and, as such, are rapidly weakened at separations much larger than the sizes of the individual constituents. As a result, chemical bond lengths are usually well below the nanometer scale. In this work, we show that molecules can *feel* each other even at very large distances if they can be efficiently coupled to a common mode of an optical cavity. By performing high-resolution spectroscopy, we investigate the modification of their energy levels and quantify the hybridization energy between them induced by the presence of the cavity mirrors. Our work sets the stage for engineering synthetic photonic states of matter.

Hybridization of molecular bonds commonly takes place when the electronic clouds of the different entities overlap (26). In addition, hybridization can occur through electrostatic couplings at very small separations (1–4), as is the case in light harvesting complexes and pigment aggregates (5–7). In this work, we demonstrate that isolated molecules at a distance can also hybridize if they couple to a common photonic mode of a high-finesse microcavity. Beyond its fundamental importance, our work has immediate implications for quantum state engineering (8, 9), and polaritonic chemistry (10–12).

When a two-level emitter is placed in a cavity, its radiative properties are modified due to the change in the density of states at its transition frequency *ω*. Within the language of cavity quantum electrodynamics (13), the coupling can be described by a cooperativity parameter $C=4g^{2}/\kappa \gamma$, where *κ* and *γ* represent the energy decay rates (linewidths) of the cavity and the emitter, respectively. Here, $g=\mu \sqrt{\omega /2\epsilon \hbar V}$ denotes the coupling strength for a cavity of mode volume *V* and emitter transition dipole moment *μ* embedded in a medium of dielectric function *ϵ*. In the weak coupling regime, the emitter radiation rate is enhanced by *C*-fold, but the photon quickly leaks out of the cavity. In the strong coupling regime, where 4g>κ+γ, energy is coherently exchanged between the emitter and the cavity mode (14, 15). These phenomena have been demonstrated both at the ensemble and single-emitter levels in a variety of contexts over the last three decades (11–13). A new challenge is to study and resolve the states that emerge from the coupling of a controlled number of emitters to a cavity, e.g., in the context of quantum state engineering and polaritonic chemistry.

## Experimental Platform

**Fig. 1.**
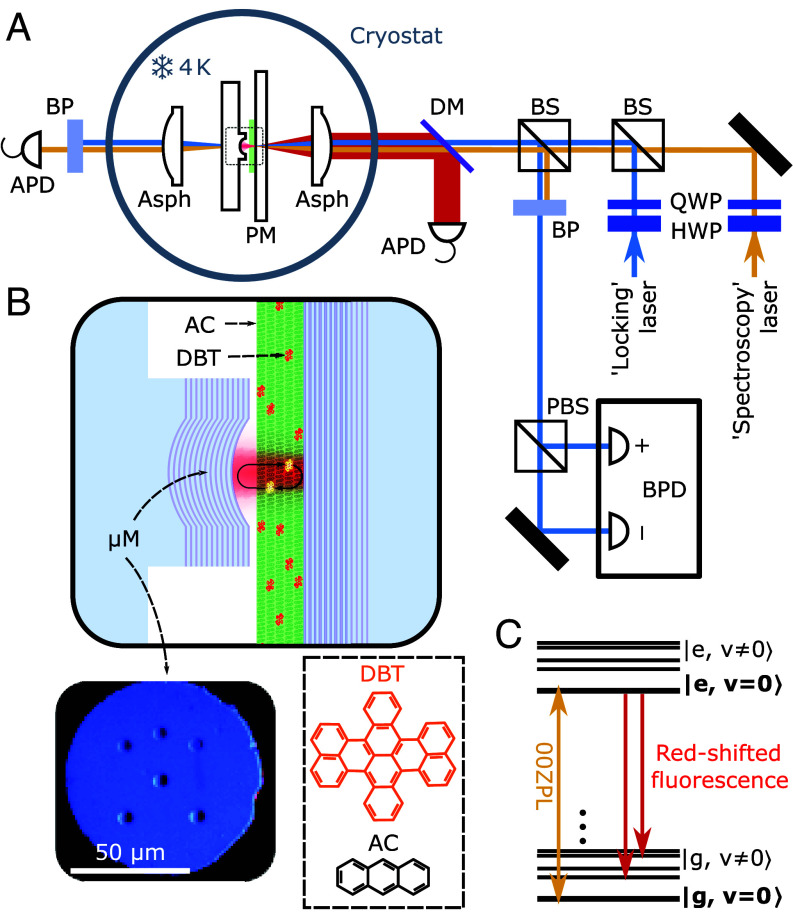
Experimental scheme. (*A*) A continuous-wave (CW) spectroscopy laser beam is coupled into the cryogenic microcavity. The transmitted laser light is detected with a photon-counting avalanche photodiode (APD). On the reflection side, we also have the possibility of detecting the red-shifted fluorescence photons with a second APD. During the measurement, a CW locking laser beam at *λ* = 700 nm is used to monitor cavity length changes. Asph: aspheric lens, DM: dichroic mirror, BP: bandpass filter, BS: nonpolarizing beam splitter, PBS: polarizing beam splitter, PM: planar mirror, QWP: quarter-wave plate, HWP: half-wave plate, and BPD: balanced photodiode. (*B*) Close-up view of the microcavity arrangement: an anthracene (AC) crystal doped with dibenzoterrylene (DBT) is placed between a flat and a curved mirror at the end of a pedestal. The curved micromirror (μM) has a radius of curvature of 10 μm. *Inset Left*: front view of a pedestal with six microfabricated curved mirrors; one is chosen for experimentation. *Inset Right*: the molecular structures of DBT and AC. (*C*) Schematics of the energy levels of a DBT molecule, indicating the vibrational manifolds of the ground and excited states.

Fig. 1*A* depicts the schematics of the experimental setup, the basic features of which were discussed in our previous publications (16, 17) (see *Materials and Methods* for details). The center piece of the experiment consists of a plano-concave Fabry–Perot microcavity that contains an anthracene (AC) crystal of submicrometer thickness extended over a lateral dimension of ∼200 μm (Fig. 1*B*). The AC crystal is doped with dibenzoterrylene (DBT) molecules, which belong to the family of polycyclic aromatic hydrocarbons and have previously been featured in many quantum and nano-optical studies (2, 16–20). DBT possesses a strong zero-phonon line (00ZPL) that connects the vibrational ground levels of the electronic ground and excited states (Fig. 1*C*). When doped in AC, the 00ZPL of DBT occurs at a wavelength of λ∼784 nm, has a Fourier-limited linewidth of γ/2π∼40 MHz at T≲4 K, and carries *α*$≃\frac{1}{3}$ of the total emission out of the excited state (denoted by branching ratio, *α*), thus, exhibiting a high degree of coherence (16, 21). As in other solid-state systems, the transition frequencies of the individual DBT molecules embedded in AC are spread over an inhomogeneous bandwidth, which was about 400 GHz in our case.

To improve the mechanical stability of the cryogenic setup beyond our previous experiments, we exploited a slight angle between the flat cavity mirror and the pedestal carrying the micromirror (Fig. 1*B*) to bring the two in gentle contact. This eliminated the need for actively locking the cavity frequency, while allowing for sufficient axial motion through actuation of a piezoelectric element to tune the cavity resonance within tens of GHz (22). Nevertheless, we used the Hänsch-Couillaud error signal obtained from the locking laser (Fig. 1*A*) to monitor the cavity stability (17). The beam from a narrow-band continuous-wave Ti:Sapph laser (spectroscopy beam) was coupled to the microcavity from the flat mirror side, and its transmission through the microcavity was recorded as the laser frequency was scanned. The data reported in this paper were collected from two cavities with different band edge frequencies for the flat distributed Bragg reflector (DBR). For the majority of the data, the red-shifted fluorescence was cut out. A full width at half-maximum (FWHM) linewidth of κ/2π=3.5 GHz on the eighth longitudinal cavity mode yielded a finesse of ∼13,500. For the two-photon measurements, the flat DBR was designed to be partially transparent for the red-shifted fluorescence emission of DBT (see *Materials and Methods* for details). In our previous works, we reported a substantial Purcell factor that led to the strong modification of the branching ratio in a single molecule (16), demonstrated the onset of single-molecule strong coupling, and presented several nonlinear optical effects at the single photon level (17). Such single-molecule studies are usually performed at low DBT:AC doping to allow spectral isolation of single molecules within the observation volume and the inhomogeneous band of DBT. In this work, we increased the DBT doping level to arrive at the regime, where the 00ZPLs of several molecules coincided with a microcavity resonance (see *Materials and Methods* for details).

## Two Molecules Coupled to a Single Cavity Mode

We start by modeling the interaction of two molecules with a single mode of a cavity. This is achieved by extending the Jaynes-Cummings Hamiltonian to the Tavis–Cummings Hamiltonian (23),

[1] $$\begin{array}{c} \begin{array}{cc} \frac{H}{\hbar } & =\omega_{1}{\sigma_{1}}^{†}\sigma_{1}+\omega_{2}{\sigma_{2}}^{†}\sigma_{2}+\omega_{c}a^{†}a\\ & +g_{1}\left(a{\sigma_{1}}^{†}+a^{†}\sigma_{1}\right)+g_{2}\left(a{\sigma_{2}}^{†}+a^{†}\sigma_{2}\right), \end{array} \end{array}$$

where $\sigma_{i}$ ($\sigma_{i}^{†}$) (i∈{1,2}) and *a* ($a^{†}$) are the lowering (raising) operators for the two emitters and the field, respectively. The parameter $\omega_{c}$ represents the cavity frequency, $\omega_{i}$ is the transition frequency of the *i*-th molecule, and $g_{i}$ describes its coupling strength to the cavity mode. In the simplified scenario of a cavity that is resonant with two identical molecules with ground and excited states $|{\text{g}}_{i}\rangle$ and $|{\text{e}}_{i}\rangle$, we obtain

[2a] $$\begin{array}{cc} \left|L\right\rangle & =\left(|{\text{g}}_{1},\;{\text{e}}_{2}\right\rangle +|{\text{e}}_{1},\;{\text{g}}_{2}\rangle )\left|0\rangle -\right|{\text{g}}_{1},\;{\text{g}}_{2}\rangle |1\rangle , \end{array}$$

[2b] $$\begin{array}{cc} \left|M\right\rangle & =\left(|{\text{g}}_{1},\;{\text{e}}_{2}\right\rangle -|{\text{e}}_{1},\;{\text{g}}_{2}\rangle )|0\rangle , \end{array}$$

[2c] $$\begin{array}{cc} \left|U\right\rangle & =\left(|{\text{g}}_{1},\;{\text{e}}_{2}\right\rangle +|{\text{e}}_{1},\;{\text{g}}_{2}\rangle )|0\rangle +|{\text{g}}_{1},\;{\text{g}}_{2}\rangle |1\rangle \end{array}$$

as new (unnormalized) polaritonic modes, where |0⟩ and |1⟩ represent the cavity field with zero and one photon, respectively. Each state can be described as a coherent superposition of three excitations. For the upper polariton state (|U⟩), all excitations are in phase, whereas for the lower polariton state (|L⟩), the molecular excitations are in phase with each other but out of phase with the photonic excitation. In the case of the middle polariton state (|M⟩), the molecular excitations are out of phase with each other, canceling the overall material dipole moment, resulting in a dark state that does not couple to the cavity mode.

**Fig. 2.**
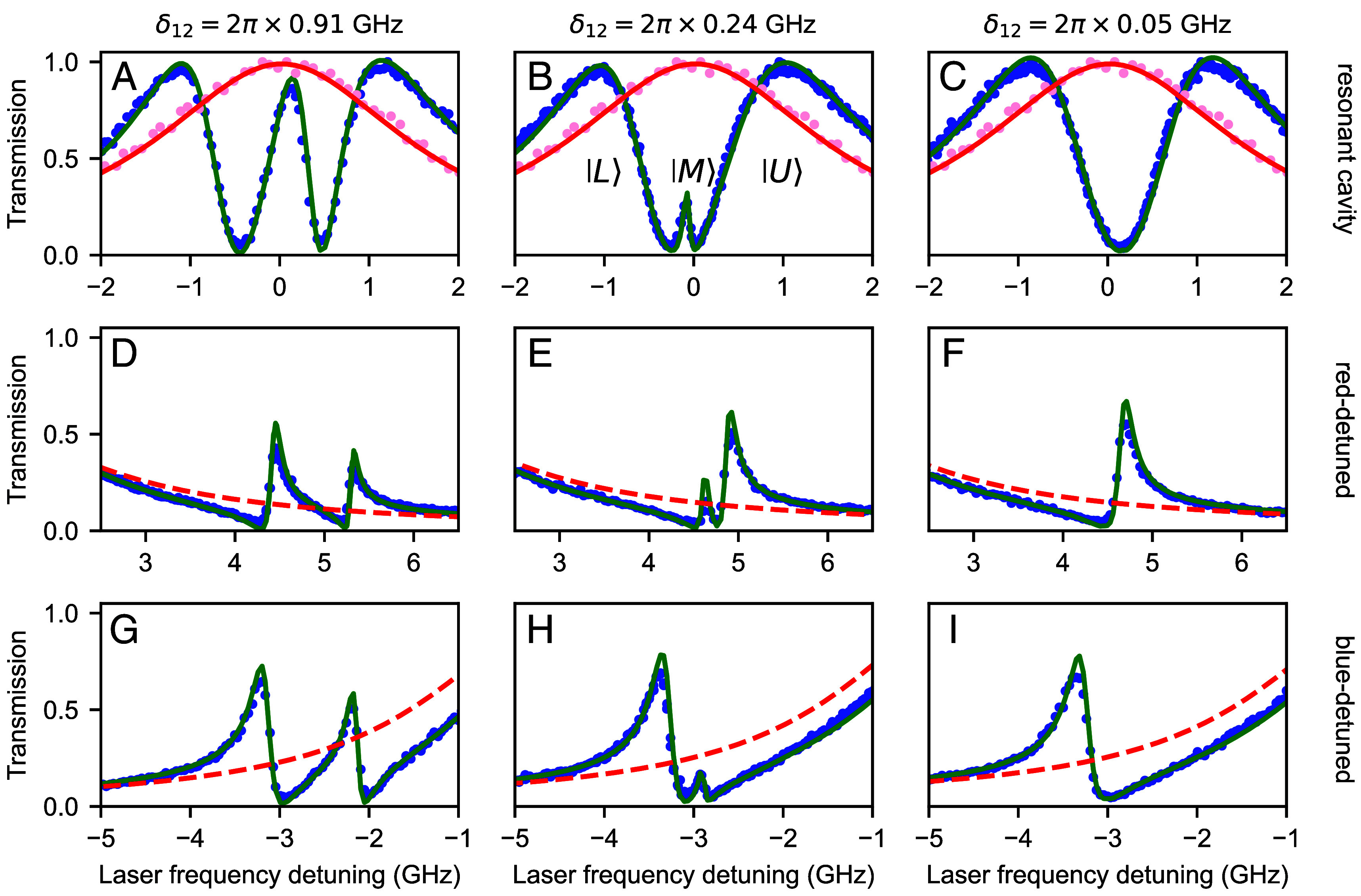
Cavity transmission when coupled to two molecules. Columns and rows group the measurements according to the frequency detuning between the two molecules and the detuning of the cavity resonance, respectively; see legends on *Top* and on the *Right*-hand side. Blue symbols show experimental data while the green curves represent the results of theoretical fits. The pink symbols and the red curve illustrate the measured spectrum of the bare cavity and a Lorentzian fit with a linewidth of 3.46 GHz, respectively. Coupling strengths were $g_{1}/2\pi =0.82\pm 0.01$ GHz and $g_{2}/2\pi =0.6\pm 0.02$ GHz. Three distinct detuning values between the molecules are presented: (*A*) $\delta_{12}/2\pi =0.91\pm 0.01$ GHz, (*B*) 0.24±0.01 GHz, and (*C*) 0.05±0.04 GHz. (*D*–*I*), show the cavity tuned to the red and blue sides, respectively. In each case, the cavity resonance is set at the origin of the horizontal axis. Red dashed curves display the extrapolated empty cavity transmission spectrum.

The red curve in Fig. 2*A* displays the fit to the cavity transmission spectrum measured far from the resonances of any molecules in the sample (25). The blue symbols show the transmission spectrum when the curved mirror was moved to tune $\omega_{c}$ to the region of $\omega_{1}$ and $\omega_{2}$, spaced by $\frac{\delta_{1,2}}{2\pi }=\frac{\omega_{1}-\omega_{2}}{2\pi }=0.91\;\pm \;0.01$ GHz. Here, $\delta_{1,2}$ is large enough that the two molecules can be considered to be independent of each other. Various cases presented in Fig. 2*B*–*I* examine the coupling for different cases of $\delta_{1,2}$ and $\Delta_{i}=\omega_{i}-\omega_{c}$.

### The Resonance Regime.

Oscillators generally couple most efficiently when they share their resonance frequencies, i.e., when $\Delta_{1}=\Delta_{2}=0$. To achieve this resonance condition, $\omega_{1,2}$ can be ideally dialed in a controlled fashion, e.g., by effectuating a local Stark effect on the DBT molecules (24). Given the absence of microelectrodes in our sample, however, we chose an alternative approach, which consisted of illuminating a molecule by a strong laser beam with an effective power of ∼1 mW in the cavity, equivalent to an intensity $I≃6\times 10^{4}$ W/cm^2^. This resulted in a frequency shift of that molecule’s 00ZPL. We attribute this effect to a slight local modification of the AC crystal in the neighborhood of the molecule caused by a small amount of energy that is deposited in it, similar to a phenomenon reported in recent publications (27, 28). Fig. 2*B* shows an example, where this approach was used to reduce $\delta_{1,2}/2\pi$ to 240 MHz. The two peaks on the right and left represent transitions to |U⟩ and |L⟩, and the small sharp feature in the middle reports on the subradiant state |M⟩ that is not fully dark.

In Fig. 2*C*, $\delta_{1,2}$ was further reduced to reach the resonance condition within 50 MHz. On resonance, the new hybridized eigenfrequencies read $\omega_{L}=\omega_{c}-\sqrt{{g_{1}}^{2}+{g_{2}}^{2}}$, $\omega_{M}=\omega_{c}$, and $\omega_{U}=\omega_{c}+\sqrt{{g_{1}}^{2}+{g_{2}}^{2}}$, respectively. Here, one only observes two peaks for transitions to |U⟩ and |L⟩ because the dark state |M⟩ does not couple to the cavity mode. In the simplest case of two identical molecules with decay rates $\gamma_{0}$ and coupling strengths $g_{0}$, the effective coupling of two molecules becomes $\sqrt{2}g_{0}$, and we obtain $\gamma_{L}=\gamma_{U}=\left(\gamma_{0}+\kappa \right)/2$. Although state |M⟩ does not couple to the cavity mode, its coupling to the remaining free-space modes yields an overall decay rate $\gamma_{M}=\gamma_{0}$. The general expressions for the decay rates $\gamma_{L},\;\gamma_{M},\;\gamma_{U}$ are presented in *SI Appendix*.

The full dynamics of the density operator is governed by a master equation (*SI Appendix*). We obtained theoretical fits (solid curves) to the measured data (symbols) presented in Fig. 2 from a numerical simulation that incorporates the coherent unitary evolution of the Tavis–Cummings Hamiltonian, the decay of the cavity mode, and the decay of molecules into free space. As $g_{1}$ and $g_{2}$ depend on *V* and on the position of the molecules within the cavity mode, they do not change if $\omega_{c}$ is varied by a small amount. This allows us to extract $g_{1}$ and $g_{2}$ from fitting the data in Fig. 2*A*. Next, we use this information to fit the spectra in Fig. 2 *B* and *C* and to deduce $\omega_{1},\omega_{2}$. The fits let us extract κ/2π=3.46±0.03 GHz, $g_{1}/2\pi =0.82\pm 0.01$ GHz and $g_{2}/2\pi =0.60\pm 0.02$ GHz. In this case, $g_{1}$ and $g_{2}$ lie just below the exceptional point of the Jaynes-Cummings Hamiltonian at $\frac{1}{4}\left(\kappa +\gamma_{0}\right)/2\pi =0.87$ GHz (29), but the collective enhancement of the vacuum Rabi splitting given by $\sqrt{g_{1}^{2}+g_{2}^{2}}/2\pi =1.02$ GHz (Fig. 2*C*) enters the regime of two-molecule strong coupling.

### The Dispersive Regime.

In the dispersive regime, where $|\Delta_{i}|\;\gg \kappa$, molecules cannot dissipate their energies into the cavity mode because the resonance condition is not met. Fig. 2*D*–*F* illustrates that if we tune the frequencies of two initially decoupled bare molecules (*D*), we first observe the formation of a partially subradiant and a bright superradiant state (*E*). Eventually, when the two molecules are resonant with each other, the subradiant state becomes fully dark (*F*) (*SI Appendix*). In other words, the two molecules hybridize via the cavity mode although they are not resonant with it. We elaborate on this in the next section. The dashed lines in the figure display the tail of the unperturbed cavity resonance, and the solid green curves present excellent theoretical fits to the experimental data. As illustrated in Fig. 2*G*–*I*, the sign of the coupling reverses if the cavity resonance is tuned to the blue side of the molecular pair. Expressions for the decay rates of the eigenstates in the dispersive regime can be found in *SI Appendix*.

## Cavity-Mediated Molecule–Molecule Coupling

The composite arrangement of two molecules in a high-finesse cavity not only strongly affects the transmission of an external laser beam, it also presents a setting in which the two molecules influence each other via the shared cavity mode. Assuming weak excitation in the dispersive regime, where the cavity is not occupied by photons, Eq. **1** simplifies (30, 31) to

[3] $$\begin{array}{c} \begin{array}{cc} \frac{H}{\hbar } & ={\tilde{\omega }}_{1}{\sigma_{1}}^{†}\sigma_{1}+{\tilde{\omega }}_{2}{\sigma_{2}}^{†}\sigma_{2}+J_{12}\left({\sigma_{1}}^{†}\sigma_{2}+\sigma_{1}{\sigma_{2}}^{†}\right). \end{array} \end{array}$$

The new frequencies ${\tilde{\omega }}_{i}=\omega_{i}+\frac{2g_{i}^{2}}{\Delta_{i}}$ and ${\tilde{\omega }}_{c}=\omega_{c}-\left(\frac{2g_{1}^{2}}{\Delta_{1}}+\frac{2g_{2}^{2}}{\Delta_{2}}\right)$ express the influence of the molecules and the cavity on each other. The coupling parameter $J_{12}=\frac{g_{1}g_{2}}{\Delta_{1}}+\frac{g_{1}g_{2}}{\Delta_{2}}$ represents the interaction energy of the two molecules through exchange of virtual photons via the common cavity mode. In analogy with the emitter–cavity interaction, one can also invoke the concept of cooperativity given by $C_{12}=\frac{4J_{12}^{2}}{\gamma_{1}\gamma_{2}}$ to describe the efficiency of the interaction between the two molecules. When including a factor of $\alpha^{2}$ to account for the branching ratios of the molecules, we obtain $C_{12}=13$ and 30 for the cases in Fig. 2 *F* and *I*, respectively.

The observed sub- and superradiant phenomena in Fig. 2*D*–*I* closely resemble the scenario of two molecules that undergo dipole–dipole coupling in the near field (2, 28, 32). The sign of the detuning Δ determines the energy ordering of the super- and subradiant states and corresponds to that of J (Δ>0) and H (Δ<0) aggregates, respectively (28, 32). Theoretical fits depicted by the solid curves in Fig. 2 *F* and *I* let us extract $J_{12}/2\pi =220\pm 10$ and −330±10 MHz, respectively. This is comparable with the dipole–dipole coupling rate of two resonant molecules separated by 25 nm (H configuration) and 28 nm (J configuration), respectively (see *Materials and Methods* for details).

**Fig. 3.**
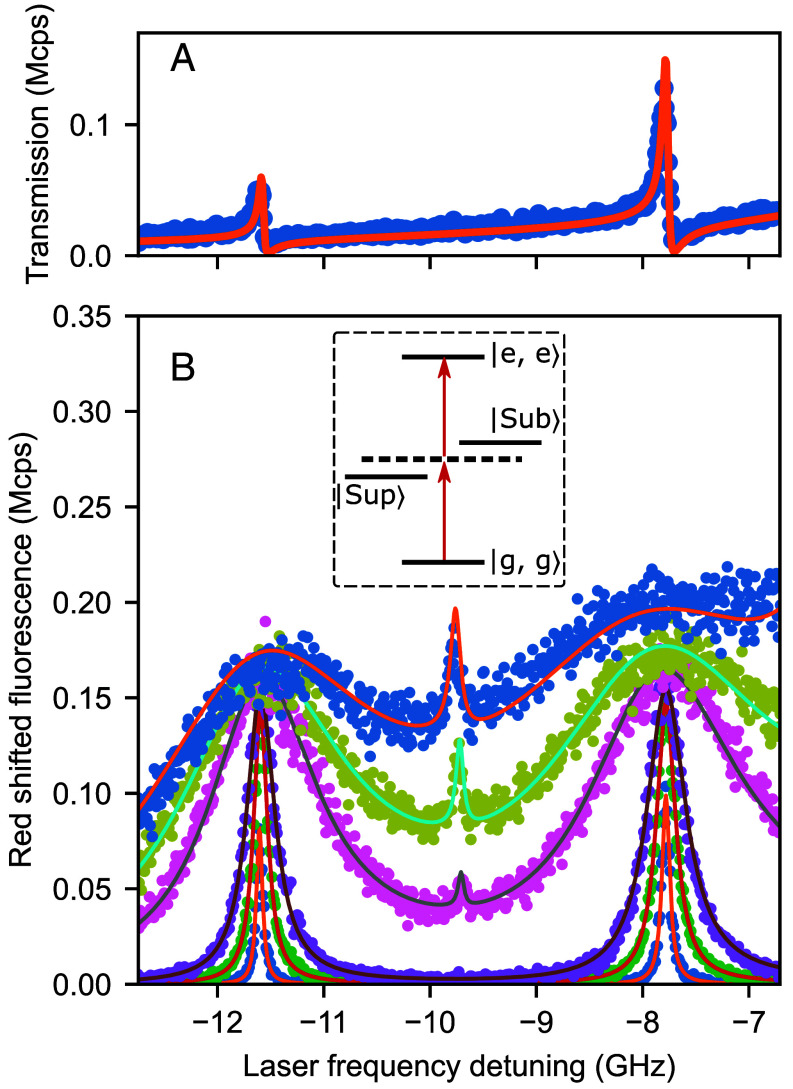
Two-photon transition. (*A*) Off-resonance transmission spectrum of the cavity in the presence of two molecules that are dispersively coupled to it. The vertical axis is expressed in the units of mega counts per second (Mcps). The x-axis is shared with (*B*). The origin marks the cavity frequency. (*B*) A series of red-shifted fluorescence spectra recorded from the cavity at increasing laser powers. At higher laser powers, a two-photon peak appears in the *Middle* of the sub- and superradiant states. The excitation powers correspond to 0.1, 0.9, 4, 80, 190, 381 photons per cavity life time, in the increasing order, respectively. *Inset* shows the energy diagram of the coupled system and the two-photon transition (red arrows). Here, the parameters were cavity linewidth κ/2π=1.58 GHz, coupling strengths $g_{1}/2\pi =0.7$ GHz and $g_{2}/2\pi =0.72$ GHz.

Previous studies have shown that an interesting consequence of the hybridization between two molecules in the near field is the emergence of a two-photon transition to the fully excited state $|e_{1},e_{2}\rangle$. This transition takes place at higher powers and appears midway between the super- and subradiant states (2, 28, 32). To investigate such a two-photon transition in our system, we increased the laser power. Fig. 3*A* shows the cavity transmission spectrum for two dispersively coupled molecules with $\delta_{12}/2\pi =3.8$ GHz, κ/2π=1.58 GHz, $g_{1}/2\pi =0.7$ GHz and $g_{2}/2\pi =0.72$ GHz (25). The symbols in Fig. 3*B* display a series of spectra recorded from the same frequency interval by recording the red-shifted fluorescence signal from the flat mirror side of the cavity. It is evident that as the laser power is increased, the original resonances become power broadened and a narrow peak appears midway. The high quality of the fits provided by the solid curves and the power dependence of the new resonance (*SI Appendix*) confirm that the new transition is a property of the new optical compound that results from the hybridization of two discrete molecules.

## Up to Eight Molecules

**Fig. 4.**
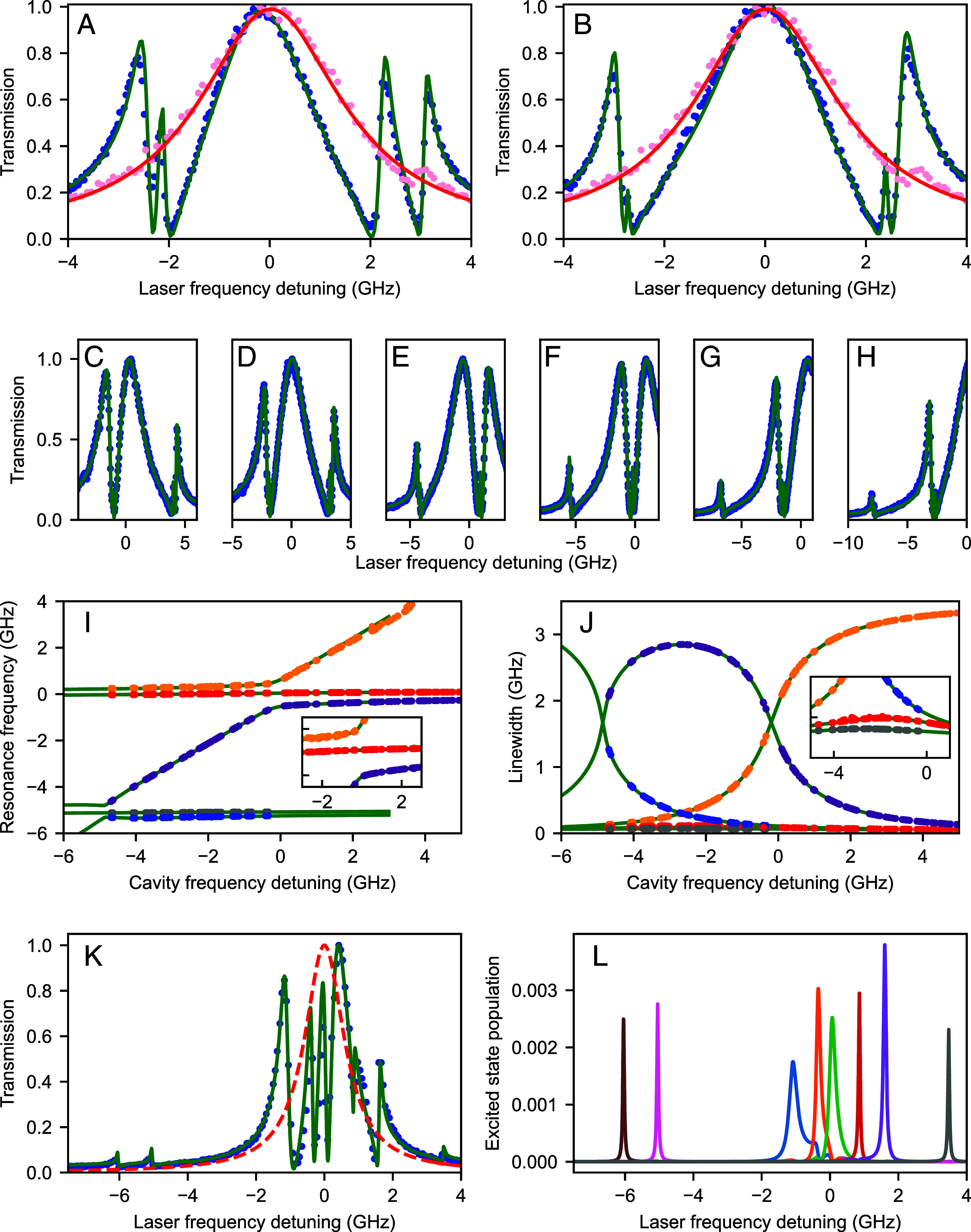
Coupling many molecules. (*A*) Two molecular pairs coupled to the cavity mode. (*B*) Same as (*A*) but with smaller frequency differences within each pair. The following parameters apply: coupling strengths $g_{1}/2\pi =0.5$ GHz, $g_{2}/2\pi =0.73$ GHz, $g_{3}/2\pi =0.82$ GHz, $g_{4}/2\pi =0.6$ GHz, and cavity linewidth κ/2π=3.46 GHz. (*C*–*H*) Snap shots of the coupled system in (*B*) for different cavity frequency detunings. (*I*) Evolution of the five resonance frequencies in (*C*–*H*). Red and gray data points represent subradiant states. *Inset* shows a close-up of the region around the origin, set to the resonance of the cavity with the blue-detuned pair. (*J*) Same as (*I*) but for resonance linewidths. *Inset* shows a close-up of the region around −3 GHz. (*K*) Symbols show the cavity transmission spectrum resulting from the coupling of eight molecules. The dashed curve shows the bare cavity resonance for comparison, placed at the origin with a linewidth of κ/2π=1.4 GHz. The solid curve presents the theoretical fit, which allows us to extract $g_{i}/2\pi =0.5,0.25,0.25,0.1,0.3$ GHz for five of the molecules. (*L*) Calculated excited-state populations of the eight molecules.

A decisive advantage of our experimental system is the relative ease with which we can increase the number of molecules that couple via a common mode of the cavity. Two key experimental features enable this. First, molecules can be doped at a fairly high level, reaching average separations that are well below 100 nm. Second, the scannable micromirror of the cavity allows one to explore different sections of the thin AC crystal, thus facilitating the identification of favorable regions in the sample. Fig. 4*A* displays the cavity transmission after dispersive coupling to four molecules, whereby one pair lies on the blue and the other on the red side of $\omega_{c}$ (25). The solid curves show the theoretical spectra fitted to the experimental data. The high fit quality lets us deduce $\Delta_{1},\;\Delta_{2},\;\Delta_{3},\;\Delta_{4}=2\pi \times$ (−2.16, −1.81, 2.18, 3.12) GHz, respectively. Fig. 4*B* displays the outcome when the frequency differences of the molecules within each pair are further reduced.

It is instructive to investigate the resonance frequencies and linewidths of the various features in the spectrum of the coupled system of Fig. 4*B* as the cavity frequency is scanned. Fig. 4*C*–*H* shows a few snap shots. In panel (*C*), the cavity is near resonant with the two red-detuned molecules, and in panel (*F*), it becomes resonant with the blue-detuned pair. Fig. 4*D*, *E*, *G*, and *H* show the cases where $\omega_{c}$ lies between the two pairs and when the cavity resonance is detuned to the blue side of both pairs, respectively. In Fig. 4*I*, we present the frequency evolution of the five resonances that result from the four molecules and the cavity as a function of $\omega_{c}$. Here, we observe an anticrossing (yellow and purple) as the cavity resonance traverses the blue-detuned pair, giving rise to the upper and lower polaritons. The central peak (red), which signifies the subradiant mode, does not experience a notable change. However, we find a small frequency shift for the superradiant states (purple and yellow; see *Inset*), which we attribute to the collective Lamb shift induced by the cavity vacuum field. A similar but weaker phenomenon takes place as $\omega_{c}$ crosses the second pair on the red-detuned side. In Fig. 4*J*, we also plot the linewidths of the various resonances. The data clearly show the broadening of the molecular resonances as they hybridize with the cavity mode to form polaritonic states. The *Inset* shows the close-up view of the slight linewidth change that occurs for the subradiant states.

The symbols in Fig. 4*K* present an example, where eight molecules are coupled to the cavity mode in the resonant or near-resonant manner. Tuning the various molecular frequencies via laser illumination was not straightforward in this case, but future integration of microelectrodes on the flat cavity mirror should allow us to explore the complex parameter space of the Dicke states (33). To gain more insight into the data, we computed the excited-state population of the individual molecules based on the outcome of the very good fit presented in Fig. 4*K*. The deviations from Lorentzian line shapes in Fig. 4*L* indicate the onset of molecule–molecule coupling.

## Discussion and Outlook

We have presented the laboratory realization of a platform, where a discrete number of molecules located at distances much larger than their physical size experience an *optical bond*. To achieve this, we coupled them to a common mode of a microcavity, entering the strong coupling regime. This paradigm is made possible by the high doping density and spectral quality of polycyclic aromatic hydrocarbons in a thin crystal coupled to a scannable Fabry–Perot microcavity. Investigation of a well-defined number of interacting emitters has also been of great interest in quantum technology and quantum optics at large, but despite several important advances (30, 34–41), scaling to large numbers has remained challenging. In this work, we showed that in addition to collective effects of the molecule-cavity system on an external laser beam, the energy levels of the individual molecules are hybridized, giving rise to sub- and superradiant states. Furthermore, we demonstrated a two-photon transition that emerges when two molecules hybridize to form a supermolecule as an optical compound.

Several improvements can be implemented in future efforts to explore and advance far-field molecular interactions. First, one can decrease the radius of curvature of the micromirror and reduce the cavity length (42) to increase the molecule-cavity coupling strength *g* by about one order of magnitude. Other resonant architectures based on photonic crystals or plasmonic nanostructures promise to offer even higher coupling strengths (38, 43–46). Moreover, integration of microelectrodes and the use of nanocrystals (47, 48) will offer more precise control of each molecular frequency and will facilitate addressing a larger number of molecules. Some of the desirable studies entail mapping the spectra of all the Dicke states that result from the coupling of *N* molecules (33, 49) and exploring new nonlinear optical phenomena (50, 51). Furthermore, collective coupling of *N* molecules can be used to enhance the effective coupling strength *g* without physical changes in the cavity design (52). This would allow, for example, to tune in the frequency of an N+1 molecule to a cavity that is resonantly coupled to *N* molecules. These advances will allow the engineering of new hybrid states of light and matter with fascinating properties (53, 54).

## Materials and Methods

### Anthracene Crystals.

We prepare DBT-doped anthracene crystals using a supersaturated vapor technique (21). First, we melt DBT and anthracene together at a concentration of 500 ppm in a sealed vial. After the mixture cools to room temperature, we transfer approximately 50 mg of it to a 50 ml pear-shaped flask which is part of the sublimation crystal growth setup shown in *SI Appendix*, Fig. S1. Then, we place a cleaned cover glass in the pear-shaped container. We evacuate and flush the flask three times with dry nitrogen, eliminating any moisture and oxygen, and in the last step, we fill it with nitrogen to 1 bar and use a 500 ^°^C heat gun to heat the flask until the powder mixture fully melts. Next, we stop heating and allow the system to cool down gradually.

As the system cools down, thin crystals begin to form on the cover glass, which we examine using a white-light cross-polarization microscope. To estimate the crystal thickness, we analyze the colors produced by birefringence of the crystal with refractive indices of $n_{a}≃1.55$ and $n_{b}≃1.78$ (55). We aim for crystal thicknesses between 400 nm and 1 μm. Additionally, we ensure the transverse size exceeds 50 μm to facilitate cavity alignment. By pressing the flat cavity mirror gently against the cover glass, some crystals are transferred. Excess crystals, which do not lie fully flat on the mirror surface, are removed by blowing nitrogen gas from a nozzle over the mirror. In the crystal used in the experiments shown in Figs. 2 and 4 of the main manuscript, we find around 40 molecules within the mode volume of the cavity. The resonance frequencies of these molecules are distributed within 150 GHz.

### Cavity Fabrication.

Our cavity consists of a flat dielectric DBR and a spherical micromirror (16, 42). The flat mirror is formed on a boro-float glass substrate measuring 10×10×0.17 mm^3^. The curved mirror is patterned at the top of a mesa structure (*SI Appendix*, Fig. S2) formed on a 0.5 mm thick fused silica. With a small mesa diameter of (100±10) μm, we achieve a cavity length in the order of 1 μm.

The mesa was fabricated via selective laser etching. Here, illumination by a femto-second pulsed laser modifies the etching rate of fused silica in KOH (56), reaching a selectivity ratio of 1:1,000 between unexposed and exposed fused silica. Once we removed the material surrounding the mesa, the plateau stood out by roughly 100 μm.

To create the concave spherical mirror on the mesa, we used focused ion beam milling in gray-scale lithography mode (42), achieving a radius of curvature of 10 μm and a central depth of 500 nm. We smoothed the edges at the outer rim to reduce scattering. The microfabricated mesa was coated with a dielectric layer stack to obtain highly reflective micromirrors with reflectivity 99.996%, matching the reflectivity of the flat DBR.

In this experiment, we constructed two types of microcavities. All experiments, except for the two-photon excitation, were performed using Bragg mirrors designed for a central wavelength of λ=785 nm with their stop-band covering large sections of the DBT emission spectrum up to 900 nm. To allow the red-shifted fluorescence of DBT to reach the detector in the measurements presented in Fig. 3 of the main manuscript, we designed the flat mirror for high reflectivity at λ=785 nm and high transmission in the range (840 to 900) nm, acting as a dichroic long-pass mirror; see *SI Appendix*, Fig. S3*A*.

The Bragg mirror consists of 25 alternating λ/4 dielectric layers, using ${\mathrm{SiO}}_{2}$ as the low-index material (n≈1.45) and ${\mathrm{Nb}}_{2}O_{5}$ as the high-index material (n≈2.25), whereby the top layer was ${\mathrm{Nb}}_{2}O_{5}$. The dichroic mirror uses alternating layers of ${\mathrm{Ta}}_{2}O_{5}$ (n≈2.07) and ${\mathrm{SiO}}_{2}$, with ${\mathrm{SiO}}_{2}$ as the topmost layer. The total dielectric coating thickness is approximately 4.85 μm. This coating is deposited on a polished fused silica substrate.

Particular care had to be taken with regard to the angular distribution of the cavity mode. With a cavity mirror radius of r=10 μm the longitudinal mode q=10 is a Gaussian standing wave with a divergence angle of *θ* = 12.7^°^ in vacuum, which amounts to *θ* = 7.2^°^ in the anthracene crystal on the flat mirror. Therefore, a considerable fraction of the energy of the mode impinges on the flat mirror at nonnormal angles of incidence (AOI), *α*. To quantify the performance of the mirrors at λ=785 nm, we used the concept of an effective reflectivity

[4] $$\begin{array}{c} R_{\text{eff}}=\int_{0}^{\pi /2}R\left(\alpha \right)P_{\theta }\left(\alpha \right)\;\text{d}\alpha , \end{array}$$

as an average of the AOI-dependent reflectivity weighted by the fraction of power $P_{\theta }\left(\alpha \right)$ propagating under *α*. Our design has an effective reflectivity of $R_{\text{eff}}=99.996$ % for the q=10 mode, very close to that of the Bragg mirrors (*SI Appendix*, Fig. S3).

### Cavity Tuning in Contact Mode.

To ensure mechanical stability without the need for active locking, we introduced a slight angular offset of approximately 5^°^ between the planar mirror and the pedestal that supports the curved micromirror. The edge of the pedestal is brought into gentle contact with the flat mirror, effectively anchoring the system and suppressing vibrations. Care is taken to ensure that the contact occurs such that the curved mirror within the pedestal (Fig. 1) is positioned directly in front of the crystal.

The contact between the pedestal edge and the crystal leaves the possibility of some axial tuning. By actuating a ring piezo, the distance between the two mirrors can be changed to within a few Å, which is sufficient to change the cavity frequency by a few tens of GHz. We point out, however, that significantly higher voltages are required to shift the cavity resonance in this contact regime, and that the standard piezo calibration factor is no longer applicable.

It can also happen that establishing contact and tuning the cavity to a given frequency within the free spectral range are not compatible. In such situations, we pursued different strategies: 1) switch to a different location on the crystal, taking advantage of potential thickness gradients, 2) switch to a different crystal, and 3) pick up a small piece of AC from the flat mirror at the edge of the pedestal. This causes the mechanical anchoring to occur slightly earlier, allowing a cavity mode that was previously only accessible in contact-free operation to now be brought into contact. If the picked-up fragment is too thick, it can be polished in situ by gently pressing it against the flat mirror while translating the mirror by a few microns.

### Laser Tuning of Molecules.

We observe shifts in the 00ZPL frequency of individual molecules following illumination with a continuous-wave (CW) laser tuned to the molecular resonance. Using a 1 mW laser (intensity of $6\times 10^{4}$ W/cm^2^), we achieve shifts of approximately 0.5 GHz within 10 s of illumination. These shifts are long-lived on experimental time scales. We note that not all molecules exhibit this behavior-some show no measurable frequency shift. We also observe that depending on the specific molecule, the tuning can occur in either direction.

In our sample, all molecules that contribute to a signal lie within the optical cavity mode and are illuminated according to its spatial profile. As a result, spatially selective excitation is not feasible. However, due to the inhomogeneous broadening of the molecular ensemble, each molecule exhibits a distinct 00ZPL frequency. We exploit this spectral inhomogeneity to address molecules in the frequency domain: The laser frequency is parked on the 00ZPL resonance of the target molecule. Although nearby molecules also see this light, only those that are near-resonant experience significant frequency shifts during the exposure time.

In both examples shown in Figs. 2 and 4, the laser frequency was resonantly parked on the initially blue-detuned molecule within each pair. In Fig. 2, from panels *A*–*C*, the blue-detuned molecule shifts by approximately −0.6 GHz, while the red-detuned molecule shifts by about +0.3 GHz. In Fig. 4, from panels *A* and *B*, the *Right*-hand pair shows a shift of −0.6 GHz for the blue-detuned molecule and +0.1 GHz for the red-detuned one. For the *Left*-hand pair, the blue-detuned molecule shifts by −0.8 GHz and the red-detuned molecule by −0.6 GHz. In all cases, the energy levels of the two molecules move closer together.

The mechanism underlying this tuning effect has been reported in previous studies on DBT molecules embedded in organic crystals. In ref. 27, laser illumination was shown to induce long-lived shifts of the molecular 00ZPLs. The effect was attributed to the formation of charge-separated states in the host matrix. In this scenario, charges generate local electric fields, resulting in Stark shifts of the molecular energy levels. More recent experiments also reported laser-induced frequency shifts, again linking the tuning effect to photoinduced charge generation (57). We did not investigate the origin of this interesting phenomenon in our laboratory but rather exploited it as a pragmatic strategy to tune molecular resonances.

### Finesse.

The full width at half-maximum (FWHM) of the cavity for the two types of microcavities mentioned above were κ=2π×3.46 GHz and κ=2π×1.58 GHz, corresponding to quality factors of Q≃109,000 and Q≃242,000 for cavities with the simple Bragg and dichroic flat mirrors, respectively. We determined the longitudinal modes of these two cavities to be q=8 and q=10, allowing us to calculate their finesse values as F≃13,500 and F≃24,000, respectively.

### Dipole–Dipole Coupling.

Two transition dipoles, $d_{1}$ and $d_{2}$, at frequencies $\omega_{0}=k_{0}c$ and decay rates *γ*, separated by a vector $r_{12}$, undergo near-field dipole–dipole coupling at the rate *J* given by (2, 58)

[5] $$\begin{array}{c} J=\frac{3\gamma }{4{\left(nk_{0}r_{12}\right)}^{3}}\left({\hat{d}}_{1}\cdot {\hat{d}}_{2}-3\left({\hat{d}}_{1}\cdot {\hat{r}}_{12}\right)\left({\hat{d}}_{2}\cdot {\hat{r}}_{12}\right)\right). \end{array}$$

For DBT molecules embedded in anthracene, the refractive index of the crystal axis *b* along which DBT molecules align is n=1.77 (59). For an *H*-aggregate ($r_{12}⊥\;d_{i}$) and a *J*-aggregate ($r_{12}\|\;d_{i}$), the coupling rates become $J_{H}=\frac{3\gamma }{4{\left(nk_{0}r_{12}\right)}^{3}}$ and $J_{J}=-\frac{3\gamma }{2{\left(nk_{0}r_{12}\right)}^{3}}$, respectively. Accounting for the branching ratio of the 00ZPL transition, for J/2π=220 MHz and J/2π=−330 MHz, corresponding to an *H*-aggregate and a *J*-aggregate, we obtain $r_{12}=25$ nm and $r_{12}=28$ nm, respectively.

### Numerical Simulations Using QUTIP (60).

We consider a cavity driven by a coherent laser at frequency $\omega_{\ell }$ and power P, resulting in a driving amplitude $\Omega =\frac{1}{2}\sqrt{\frac{\kappa P}{\hbar \omega_{\ell }}}$. In a frame rotating at the laser frequency, the system’s Hamiltonian is given by

[6] $$\begin{array}{c} \begin{array}{cc} \frac{H}{\hbar } & =\Delta_{1}\sigma_{1}^{†}\sigma_{1}+\Delta_{2}\sigma_{2}^{†}\sigma_{2}+\Delta_{c}a^{†}a\\ & +g_{1}\left(a\sigma_{1}^{†}+a^{†}\sigma_{1}\right)+g_{2}\left(a\sigma_{2}^{†}+a^{†}\sigma_{2}\right)+\Omega \left(a+a^{†}\right), \end{array} \end{array}$$

where $\Delta_{i}=\omega_{i}-\omega_{\ell }$ and $\Delta_{c}=\omega_{c}-\omega_{\ell }$ are the detunings of the *i*th emitter and the cavity resonance from the laser frequency, respectively. The last term in Eq. **6** represents the coherent laser drive. We note that the notation $\Delta_{i}$ in this passage is different from that used in the main text. The measured spectra are modeled through numerical simulations based on the Liouvillian superoperator formalism (*SI Appendix*). These simulations incorporate both the coherent dynamics governed by the two (or many)-emitter Tavis–Cummings Hamiltonian, including the coherent laser drive, and the dissipative processes, such as cavity losses and the free-space decay of the emitters.

Using this approach, we compute the steady-state density matrix $\rho (\omega_{\ell })$ for each laser frequency. The transmitted light, proportional to the steady-state cavity intensity, is given by

$$n\left(\omega \right)=\mathrm{Tr}(a^{†}a\rho \left(\omega_{\ell }\right)),$$

while the population of the excited state of the *i*th emitter reads

$$\rho_{\mathrm{ee}}^{i}\left(\omega \right)=\mathrm{Tr}\left(\sigma_{i}^{†}\sigma_{i}\rho \left(\omega_{\ell }\right)\right).$$

All fits shown in Figs. 2, 3*A*, and 4*A*, *B*, *K*, and *L* of the main manuscript were obtained using this methodology. Using this approach, we compute the population distributions for the 2- and 4-molecule cases as presented in *SI Appendix*, Figs. S4 and S5, respectively.

### Extracting Linewidths and Central Frequencies.

In the regime illustrated in Fig. 4, the cavity mode hybridizes with four molecular resonances, resulting in five new eigenstates. Each of these modes inherits a distinct admixture of cavity and molecular character depending on the detuning and coupling strength. To determine the central frequencies and linewidths of these eigenstates, we fit the spectral data using a model consisting of five independent Fano profiles and a constant offset term. The Fano resonance, which describes the characteristic asymmetric line shapes, is mathematically expressed as

[7] $$\begin{array}{c} F\left(\nu ,A,\nu_{0},\sigma ,q\right)=A\frac{{\left[\left(\nu -\nu_{0}\right)+\frac{q\sigma }{2}\right]}^{2}}{{\left(\frac{\sigma }{2}\right)}^{2}+{\left(\nu -\nu_{0}\right)}^{2}}. \end{array}$$

Here, *ν* represents the laser frequency, *A* corresponds to the amplitude of the resonance, the central frequency of the resonance is denoted by $\nu_{0}$, while *σ* represents the linewidth, and the degree of asymmetry in the line shape is characterized by the Fano parameter *q*, which is treated as an independent fitting parameter for each resonance.

The sum of the five Fano profiles and the offset is simultaneously fit to the measured spectrum. This fitting approach effectively captures the spectral features. The extracted central frequencies and linewidths provide insights into the coupling dynamics between the cavity mode and the molecular resonances. This methodology was applied to fit the spectra presented in Fig. 4*C*–*H* and all spectra contributing to Fig. 4 *I* and *J*.

## Supplementary Material

Appendix 01 (PDF)

## Data Availability

The data and plotting scripts used to generate all figures in this study are publicly available at Zenodo: https://doi.org/10.5281/zenodo.15575243 (25).
