# Supplementary material for: Hybridization of molecules via a common photonic mode
Source: Proc Natl Acad Sci U S A. 2025 Jul 30;122(31):e2505161122. doi: 10.1073/pnas.2505161122 (PMC12337323; doi:10.1073/pnas.2505161122)
Supplement: Supplementary file 1 — Appendix 01 (PDF) [file pnas.2505161122.sapp.pdf]

Supporting Information for

Hybridization of molecules via a common photonic mode

Jahangir Nobakht, André Pscherer, Jan Renger, Stephan Götzinger, and Vahid Sandoghdar

Vahid Sandoghdar.
E-mail: vahid.sandoghdar@mpl.mpg.de

This PDF file includes:

Supporting text
Figs. S1 to S6
SI References

Supplementary figures

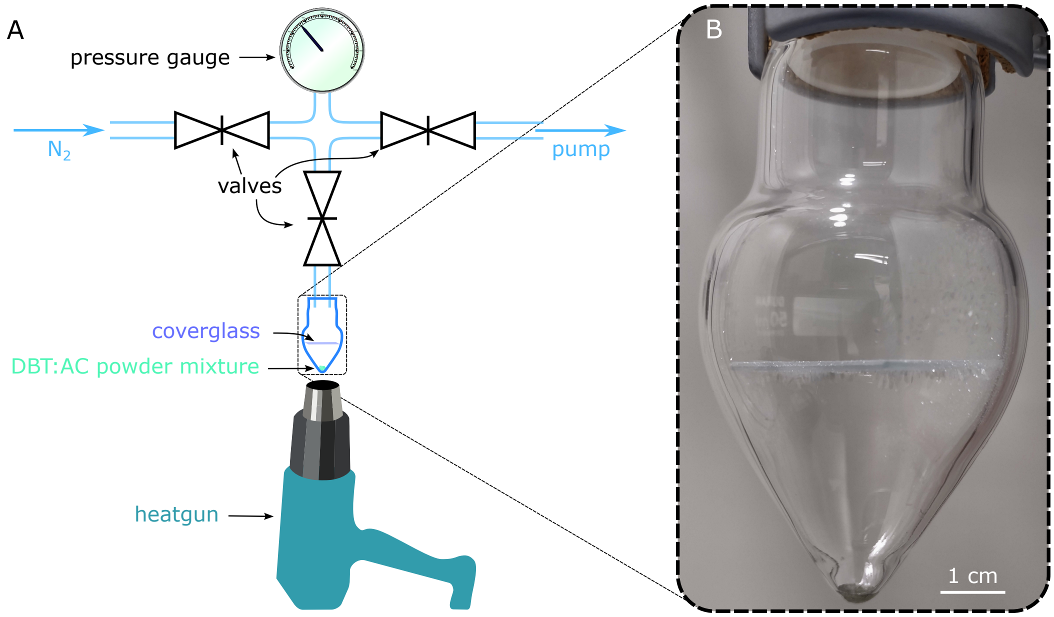

Fig. S1. Crystal growth. (A) Setup for anthracene crystal growth. (B) Close-up of the flask with the coverglass after the crystals have grown.

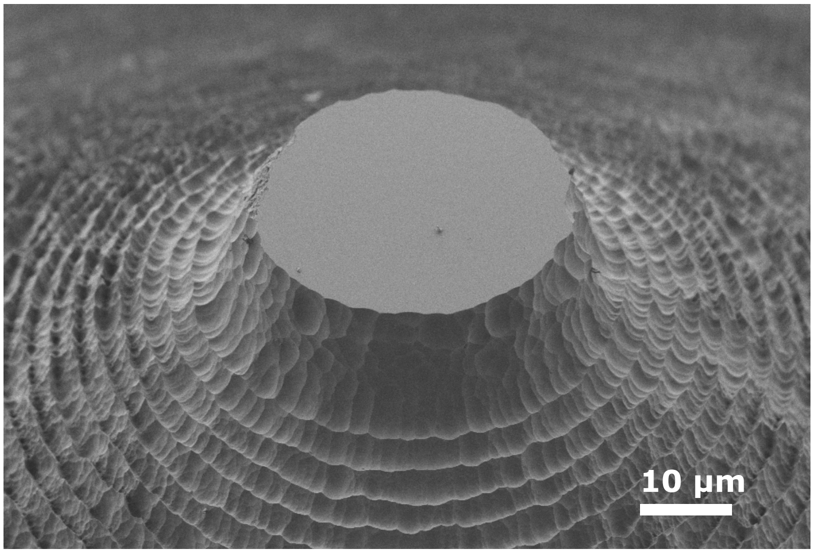

Fig. S2. Microfabricated pedestal. Scanning electron microscope (SEM) image of a microfabricated pedestal.

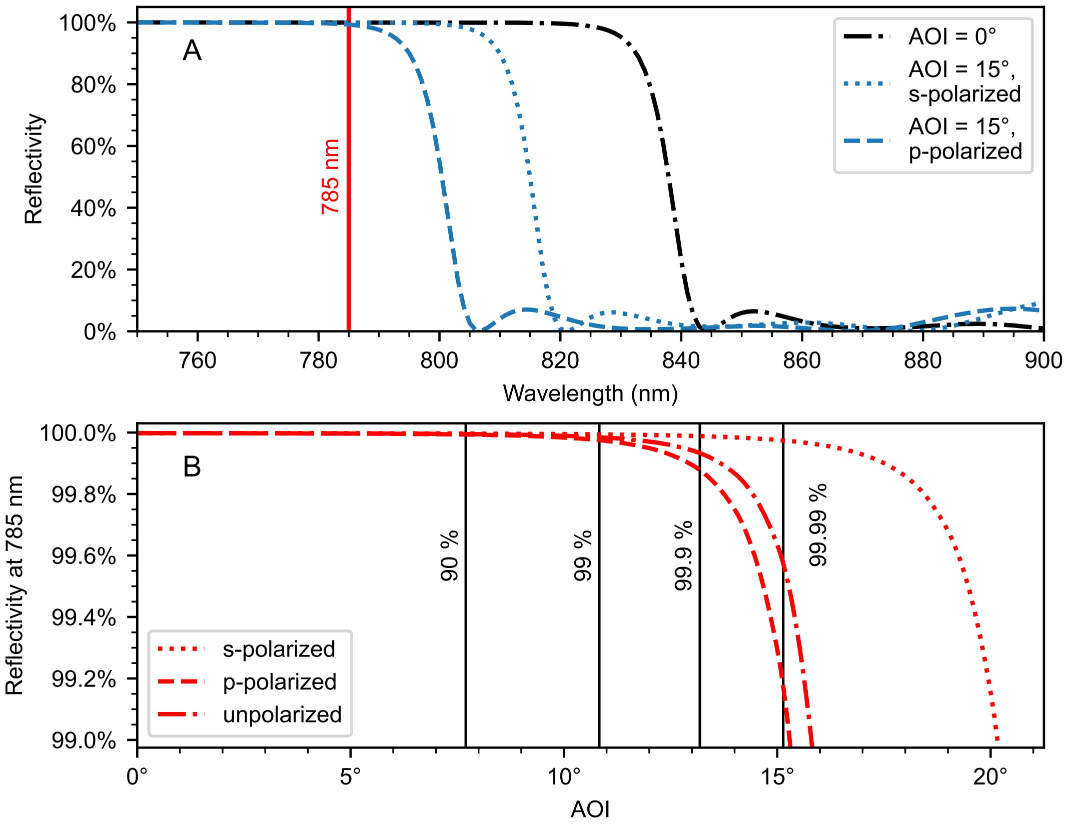

Fig. S3. Reflectivity spectrum and angular dependence. (A) Reflectivity spectrum of the dichroic cavity mirrors for AOI=$0\,^\circ$ and $15\,^\circ$. (B) Reflectivity as a function of AOI. Vertical lines indicate the fraction of power contained in a beam of divergence $\theta = 7.2\,^\circ$ up to their corresponding AOI.

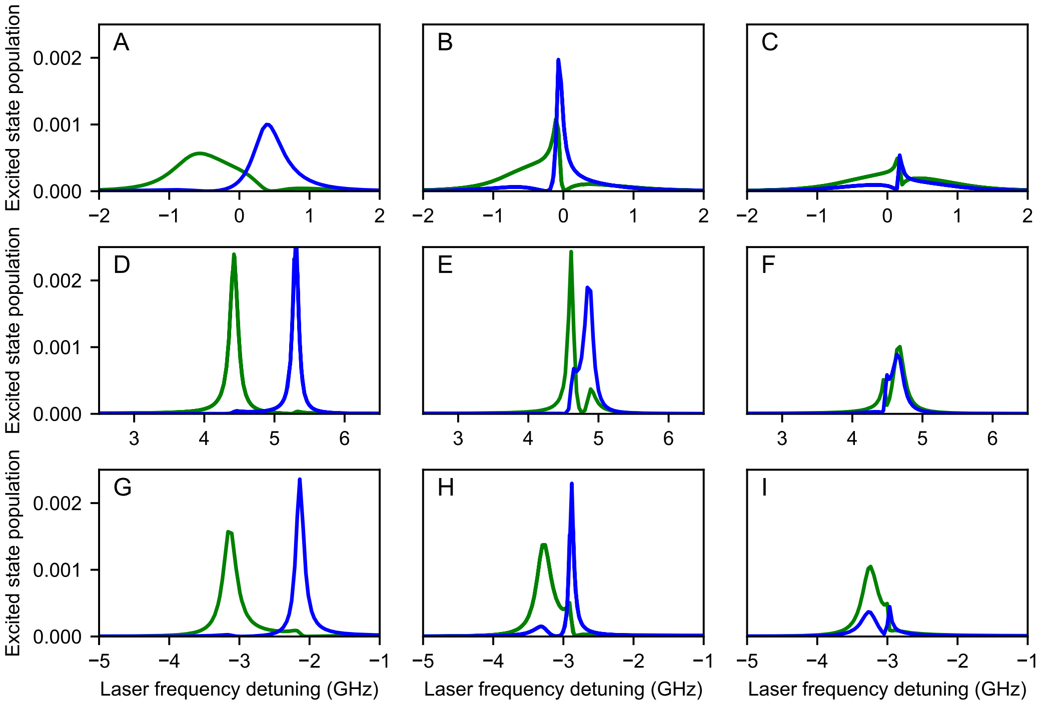

Fig. S4. Excited State Populations of two Molecules. (A–I), Populations of the excited states of the two molecules, as illustrated in Fig. 2(A–I) of the main manuscript.

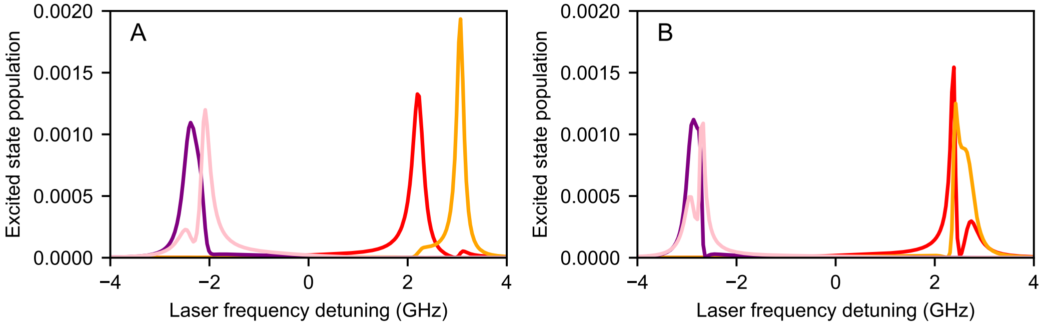

Fig. S5. Excited State Populations of Four Molecules. (A, B) Populations of the excited states of the four molecules, as shown in Fig. 2(A, B) of the main manuscript.

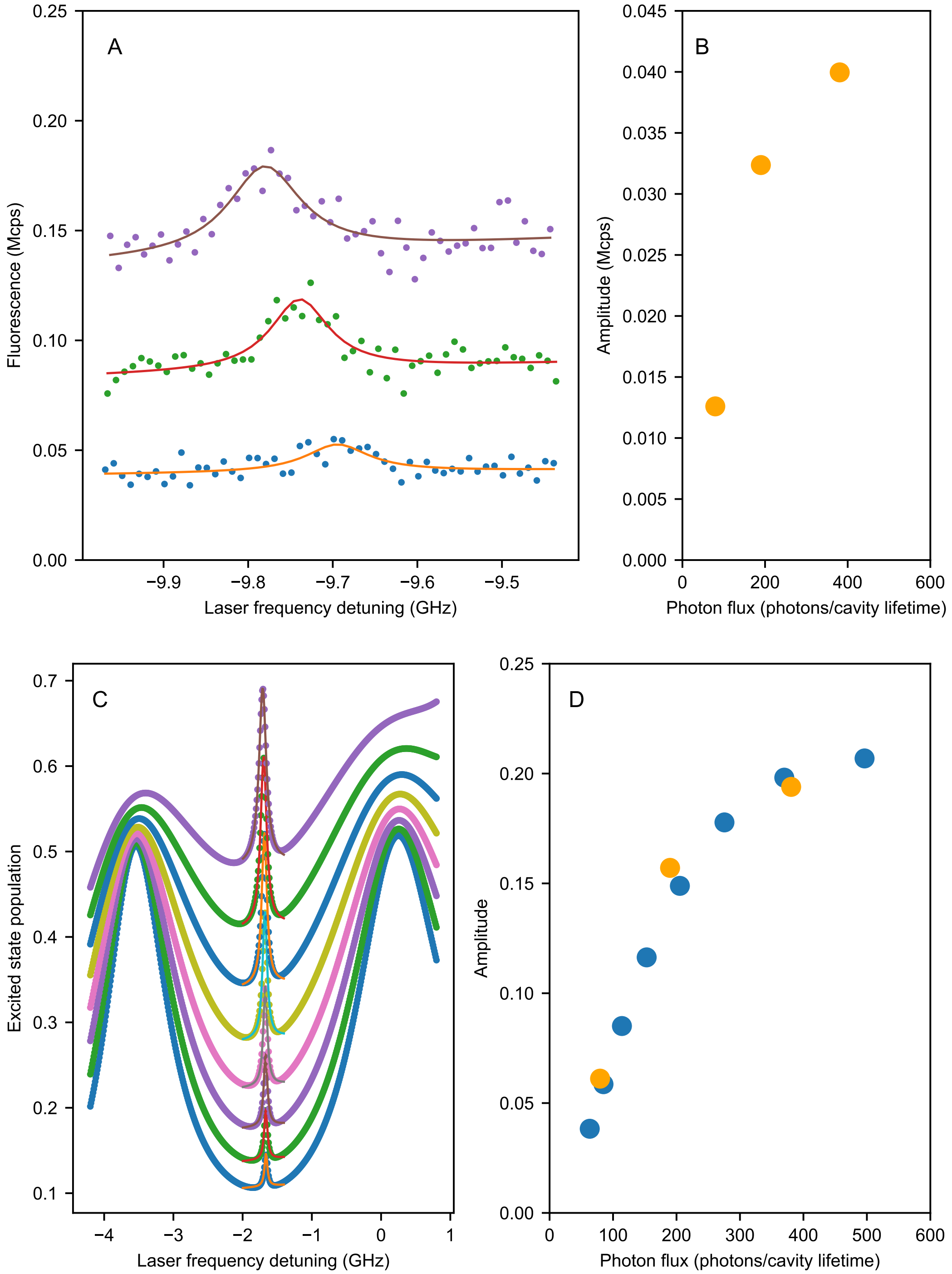

Fig. S6. Two photon peak power dependence. (A) Fluorescence spectra with Lorentzian fits for three independent measurements. (B) Amplitude of the Lorentzian peaks in a, plotted as a function of photon flux. (C) Theoretical simulations extending the power range with finer steps. (D) Amplitudes of the Lorentzian fits from (C), plotted as a function of photon flux, illustrating theoretical predictions, with the experimental data points from (B) overlaid for comparison.

Supporting Information Text

Theoretical model. We consider two molecules modeled as two-level quantum emitters with electronic ground and excited states $|\mathsf{g}_i\rangle$ and $|\mathsf{e}_i\rangle$ and resonance frequencies ω_i $(i \in \{1,2\})$. These emitters have a transition dipole moment μ and interact with a single mode of the cavity field at frequency ω_c. The coupling constant (at field maximum) is given by

$$g = \mu\sqrt{\omega/2\epsilon\hbar V}, \qquad [1]$$

where V denotes the optical mode volume, and ϵ is the permittivity. This system can be modeled by a Tavis-Cummings Hamiltonian which describes the coherent exchange of excitation between the cavity mode and each emitter,

$$\begin{aligned}\frac{\mathcal{H}}{\hbar} &= \omega_1{\sigma_1}^\dagger\sigma_1 + \omega_2{\sigma_2}^\dagger\sigma_2 + \omega_c a^\dagger a \\ &+ g_1(a{\sigma_1}^\dagger + a^\dagger\sigma_1) + g_2(a{\sigma_2}^\dagger + a^\dagger\sigma_2).\end{aligned} \qquad [2]$$

The operators σ_i $(\sigma_i^\dagger)$ $(i \in \{1,2\})$ and a $(a^\dagger)$ are emitter and field lowering (raising) operators, respectively. The full dynamics of the density operator is governed by the master equation,

$$\frac{d\rho}{dt} = i\left[\frac{\mathcal{H}}{\hbar},\rho\right] + \mathcal{L}_\kappa[\rho] + \mathcal{L}_{\gamma_1}[\rho] + \mathcal{L}_{\gamma_2}[\rho], \qquad [3]$$

which accounts for dissipation and decoherence of the emitter and the cavity field due to their interactions with the vacuum modes of the open system via Lindblad terms. The Lindblad superoperators are given by,

$$\mathcal{L}_\kappa[\rho] = \kappa\left(a\rho a^\dagger - \frac{1}{2}\{a^\dagger a,\rho\}\right), \qquad [4a]$$

$$\mathcal{L}_{\gamma_i}[\rho] = \gamma_i\left(\sigma_i\rho{\sigma_i}^\dagger - \frac{1}{2}\{{\sigma_i}^\dagger\sigma_i,\rho\}\right). \qquad [4b]$$

The notation [...] is used for the argument of the superoperators, $\{O,\rho\} = O\rho + \rho O$. Furthermore, κ is the photon number decay rate, and γ is the spontaneous emission rate of the quantum emitter.

Resonant regime. For a resonant emitter-cavity system in the weak excitation regime, we can focus on the Hamiltonian in the single excitation subspace spanned by $|\mathsf{g}_1,\mathsf{g}_2,1\rangle$, $|\mathsf{e}_1,\mathsf{g}_2,0\rangle$ and $|\mathsf{g}_1,\mathsf{e}_2,0\rangle$, which reads

$$\frac{\mathcal{H}}{\hbar} = \begin{pmatrix} \omega_c & g_1 & g_2 \\ g_1 & \omega_1 & 0 \\ g_2 & 0 & \omega_2 \end{pmatrix}. \qquad [5]$$

When both emitters and the cavity are resonant ($\omega_1 = \omega_2 = \omega_c := \omega$), $\mathcal{H}$ has eigenstates $|L\rangle$, $|M\rangle$ and $|U\rangle$ with the corresponding annihilation operators

$$\sigma_L = \frac{1}{\sqrt{2(g_1^2+g_2^2)}}(g_1\sigma_1 + g_2\sigma_2 - \sqrt{g_1^2+g_2^2}\,a), \qquad [6a]$$

$$\sigma_M = \frac{1}{\sqrt{g_1^2+g_2^2}}(g_1\sigma_2 - g_2\sigma_1), \qquad [6b]$$

$$\sigma_U = \frac{1}{\sqrt{2(g_1^2+g_2^2)}}(g_1\sigma_1 + g_2\sigma_2 + \sqrt{g_1^2+g_2^2}\,a). \qquad [6c]$$

For the σ_M transition, the field emitted by molecule 1 ($\sim \sigma_1$) is out of phase with that of molecule 2 ($\sim \sigma_2$). For the σ_L transition, the fields emitted by the two molecules are in phase while the field of the cavity is out of phase with them. For the σ_U transitions, the fields emitted by the two molecules as well as the cavity field are all in phase.

Utilizing these transformations, we can diagonalize the Hamiltonian to arrive at

$$\begin{aligned}\frac{\mathcal{H}}{\hbar} &= \omega\sigma_M^\dagger\sigma_M \\ &+ (\omega - \sqrt{{g_1}^2+{g_2}^2})\sigma_L^\dagger\sigma_L \\ &+ (\omega + \sqrt{{g_1}^2+{g_2}^2})\sigma_U^\dagger\sigma_U\,,\end{aligned} \qquad [7]$$

which yields three decoupled modes $|L\rangle$,$|M\rangle$ and $|U\rangle$ at frequencies $\omega - \sqrt{{g_1}^2+{g_2}^2}$, ω, and $\omega + \sqrt{{g_1}^2+{g_2}^2}$, corresponding to the lower polariton, middle resonance, and upper polariton, respectively. The two polaritons (L and U) are entangled light-matter states separated by $2\sqrt{{g_1}^2+{g_2}^2}$. The decay rates (linewidths) of the three resonances are obtained by writing the Lindblad terms in the new basis:

$$\gamma_M = \frac{\gamma_2 g_1^2 + \gamma_1 g_2^2}{g_1^2+g_2^2}, \qquad [8a]$$

$$\gamma_L = \gamma_U = \frac{\gamma_1 g_1^2 + \gamma_2 g_2^2}{2\,(g_1^2+g_2^2)} + \frac{\kappa}{2}. \qquad [8b]$$

Next, by introducing a detuning ($\delta = \omega_2 - \omega_1$) between the two emitters while keeping the cavity frequency at the center, we obtain modified eigenstates. In the particular case of the middle resonance and under the assumption that $g_1 \simeq g_2 := g$ we obtain,

$$\sigma_M = \frac{1}{\sqrt{2g^2 + \delta^2}}(g\sigma_2 - g\sigma_1 + \delta a), \tag{9a}$$

$$\gamma_M = \frac{\delta^2\kappa + (\gamma_1 + \gamma_2)\, g^2}{\delta^2 + 2g^2}\,. \tag{9b}$$

Equation Eq. (9a) illustrates the contribution of the cavity mode to the middle resonance (M), which diminishes as δ approaches zero. Similarly, equation Eq. (9b) highlights the role of the cavity linewidth (κ) in broadening the linewidth of the middle resonance.

Dispersive regime. In the dispersive regime, where $|\omega_c - \omega_i| \gg \kappa$, and assuming weak excitation such that the cavity is not occupied with photons, the Hamiltonian introduced in Eq. Eq. (2) simplifies to

$$\frac{\mathcal{H}}{\hbar} = \tilde{\omega}_1\sigma_1^\dagger\sigma_1 + \tilde{\omega}_2\sigma_2^\dagger\sigma_2 + J(\sigma_1^\dagger\sigma_2 + \sigma_1\sigma_2^\dagger). \tag{10}$$

In this context, $\tilde{\omega}_i = \omega_i + \frac{2g_1^2}{\omega_1 - \omega_c}$ signifies the Lamb-shifted emitter frequency, $\tilde{\omega}_c = \omega_c - \frac{2g_1^2}{\omega_1 - \omega_c} - \frac{2g_2^2}{\omega_2 - \omega_c}$ denotes the modification of the cavity frequency, and $J = \frac{g_1 g_2}{\Delta_1} + \frac{g_1 g_2}{\Delta_2}$ represents the rate of the coupling between the two molecules mediated by the cavity. For single molecules coupled to the cavity, the decay rate into the cavity mode is given by

$$\tilde{\gamma}_i = \frac{4g_i^2}{\kappa}\frac{\left(\frac{\kappa}{2}\right)^2}{(\omega_i - \omega_c)^2 + \left(\frac{\kappa}{2}\right)^2}. \tag{11}$$

In the single-excitation subspace, the eigenstates of the coupled emitters become hybridized as

$$|-\rangle = \cos\theta|\mathsf{e}_1\mathsf{g}_2\rangle - \sin\theta|\mathsf{g}_1\mathsf{e}_2\rangle, \tag{12a}$$

$$|+\rangle = \sin\theta|\mathsf{e}_1\mathsf{g}_2\rangle + \cos\theta|\mathsf{g}_1\mathsf{e}_2\rangle, \tag{12b}$$

whereby the mixing angle θ is determined by

$$\tan 2\theta = \frac{2J}{\tilde{\omega}_2 - \tilde{\omega}_1}. \tag{13}$$

These hybridized states exhibit distinct frequencies,

$$\omega_- = \tilde{\omega}_1\sin^2\theta + \tilde{\omega}_2\cos^2\theta + 2J\sin\theta\cos\theta, \tag{14}$$

$$\omega_+ = \tilde{\omega}_1\cos^2\theta + \tilde{\omega}_2\sin^2\theta - 2J\sin\theta\cos\theta. \tag{15}$$

The decay rates into the cavity mode are expressed as (1, 2),

$$\gamma_+ = \tilde{\gamma}_1\sin^2\theta + \tilde{\gamma}_2\cos^2\theta + 2\gamma_{12}\sin\theta\cos\theta, \tag{16}$$

$$\gamma_- = \tilde{\gamma}_1\cos^2\theta + \tilde{\gamma}_2\sin^2\theta - 2\gamma_{12}\sin\theta\cos\theta. \tag{17}$$

Here, $\gamma_{12} = \sqrt{\tilde{\gamma}_1\tilde{\gamma}_2}$ represents the cross-damping term. In the special case where $\omega_1 = \omega_2 := \omega$ and $g_1 = g_2 := g$, leading to $\tilde{\omega}_1 = \tilde{\omega}_2$, the mixing angle becomes $\theta = \pi/4$. In this scenario, the states $|+\rangle$ and $|-\rangle$ are maximally entangled (3). The decay rates into the cavity mode simplify to

$$\gamma_+ = \frac{(\sqrt{\tilde{\gamma}_1} + \sqrt{\tilde{\gamma}_2})^2}{2}, \tag{18}$$

$$\gamma_- = \frac{(\sqrt{\tilde{\gamma}_1} - \sqrt{\tilde{\gamma}_2})^2}{2} = 0, \tag{19}$$

corresponding to superradiant and subradiant decay paths (3).

Two-photon peak. To quantify the observed two-photon resonance peaks in Fig. 3B of the main manuscript, we fit them using a Lorentzian function combined with a linear background. The Lorentzian function captures the resonance profile, while the linear background accounts for any underlying baseline trend. The fitting was performed using the following function:

$$L(x) = a + b\cdot x + \frac{c}{1 + \left(\frac{x - x_0}{\gamma}\right)^2}. \tag{20}$$

Figure S6A illustrates the fluorescence spectra for three measurements, overlaid with their corresponding fits. Figure S6B presents the extracted Lorentzian amplitudes (c) as a function of the excitation photon flux. The photon flux values were assessed considering the cavity lifetime and the input power. This plot highlights the nonlinear behavior of the two-photon resonance.

Estimation of Near-Field Coupling Probability. When increasing the density of guest molecules in the host crystal, at some point one expects the neighboring molecules to become close enough to experience near-field dipole-dipole coupling, i.e., if their resonance frequencies are also reasonably similar. To assess the likelihood of our sample being in this regime, we estimate the joint probability of spatial and spectral proximity that would enable such near-field interactions.

The mode volume of a Gaussian beam standing wave is

$$V = \frac{\pi}{4} w_0^2 L.$$

Taking $w_0 = 1.1\,\mu\text{m}$ and L as the thickness of the crystal ($\sim 500\,\text{nm}$), the volume is approximately $0.5\,\mu\text{m}^3$. From the observation of approximately 40 molecules within the cavity mode volume, we infer a molecular density of

$$\rho = \frac{40}{0.5\,\mu\text{m}^3} = 80\,\mu\text{m}^{-3}.$$

The average number of neighboring molecules within a radius r of a given molecule is given by:

$$n = \frac{4}{3}\pi\rho r^3.$$

For a critical distance of $r = 50\,\text{nm} = 0.05\,\mu\text{m}$, where the dipole–dipole interaction strength J becomes comparable to the radiative decay rate γ, we find:

$$n = \frac{4}{3}\pi \cdot 80\,\mu\text{m}^{-3} \cdot (0.05\,\mu\text{m})^3 \approx 0.042.$$

For a given pair of molecules with low enough detuning (as the pairs investigated in this paper), the probability that their distance is below 50 nm is:

$$\frac{\frac{4}{3}\pi(50\,\text{nm})^3}{\frac{\pi}{4} w_0^2 L} = 0.0011.$$

Next, we estimate the spectral overlap probability. Assuming the inhomogeneous broadening of the molecular transitions is uniformly distributed across a 150 GHz bandwidth, the probability that two molecules are within a spectral detuning of ± 1 GHz is:

$$P_\text{spec} = \frac{2\,\text{GHz}}{150\,\text{GHz}} = \frac{1}{75}.$$

Combining spatial and spectral considerations, the total probability p that a given molecule has at least one neighbor within both the spatial and spectral conditions required for near-field coupling is:

$$p = n \times P_\text{spec} \approx 0.042 \times \frac{1}{75} \approx 5.6 \times 10^{-4}.$$

This low probability indicates that under our experimental conditions, direct dipole–dipole interactions are exceedingly rare.

References

1. C Hettich, et al., Nanometer Resolution and Coherent Optical Dipole Coupling of Two Individual Molecules. *Science* **298**, 385–389 (2002).
2. U Akram, Z Ficek, S Swain, Decoherence and coherent population transfer between two coupled systems. *Phys. Rev. A* **62**, 013413 (2000).
3. RH Dicke, Coherence in Spontaneous Radiation Processes. *Phys. Rev.* **93**, 99–110 (1954).
